# APC-rearranged solid pseudopapillary neoplasm–pancreatic neuroendocrine tumor collision tumor: a molecularly resolved case report

**DOI:** 10.3389/fonc.2026.1796302

**Published:** 2026-05-07

**Authors:** Jian Guan, Yin Lu, Huijuan Zhang, Wenting Huang

**Affiliations:** Department of Pathology, National Cancer Center/National Clinical Research Center for Cancer/Cancer Hospital & Shenzhen Hospital, Chinese Academy of Medical Sciences and Peking Union Medical College, Shenzhen, China

**Keywords:** APC, case report, collision tumor, CTNNB1, PanNET, SPN

## Abstract

**Background:**

Solid-pseudopapillary neoplasm (SPN) is driven by CTNNB1 mutations, whereas pancreatic neuroendocrine tumors (PanNETs) harbor MEN1/DAXX/ATRX alterations. A true collision of the two entities has never been genetically proven.

**Case presentation:**

A 34-year-old man presented with a 4.2 cm pancreatic-tail mass and innumerable liver metastases. Distal pancreatectomy disclosed intimately admixed yet distinct SPN (90%) and G1 PanNET (10%). Targeted NGS revealed an in-frame APC rearrangement (Ex9-Int15); CTNNB1, MEN1, DAXX and ATRX were wild-type.

**Outcome:**

Following distal pancreatectomy and radiofrequency ablation of liver metastases, the patient remains asymptomatic with no evidence of disease progression at 9-month follow-up.

**Discussion:**

This is the first molecularly confirmed SPN–PanNET collision tumor driven by APC loss rather than CTNNB1 mutation. The case highlights the value of comprehensive genomic profiling when morphologic overlap obscures diagnosis and suggests that APC-driven SPNs may be more aggressive than previously thought.

## Background

Solid-pseudopapillary neoplasm (SPN) is an uncommon pancreatic tumor of low malignant potential that almost always harbors activating CTNNB1 mutations, resulting in diffuse nuclear β-catenin accumulation and constitutive Wnt pathway activation (1, 2). Pancreatic neuro-endocrine tumors (PanNETs), in contrast, retain membranous β-catenin and are driven by MEN1, DAXX/ATRX, or mTOR-pathway alterations (3). The synchronous coexistence of SPN and PanNET is extraordinarily rare and is generally interpreted as a “collision tumor” rather than divergent differentiation from a shared precursor.

Since 2019, an additional molecular subset of morphologically classic SPNs that lack CTNNB1 mutations has been recognized (4). These tumors instead show inactivation of the APC tumor-suppressor gene, either through frameshift deletion or genomic rearrangements. By pooling all previously reported APC-driven SPNs with our present case, we show that—although their histology and immunophenotype mirror those of conventional CTNNB1-mutant tumors—they constitute a clinicopathologically distinct entity with unique demographics, disease distribution, and clinical behavior.

We therefore describe a patient with co-existing APC-rearranged SPN and low-grade PanNET (G1), emphasize the diagnostic challenges posed by morphologically overlapping components, and underscore the importance of generous sampling and comprehensive genomic profiling for accurate classification and optimal patient management.

## Case presentation

### Patient information

A 34-year-old man with no significant medical history was incidentally found to have multiple hepatic masses and a pancreatic tail lesion during a routine health screening. He denied abdominal pain, nausea, weight loss, or constitutional symptoms. Family history was notable for cervical carcinoma in his mother and lung carcinoma in his maternal grandfather. His medical history was listed in **Table 1**.

**Table 1:** The patient’s clinical history.

Timeline	Key event/intervention	Main findings	Outcome
Nov 2022	Routine check-up → imaging abnormality found	4.2-cm pancreatic-tail mass + multiple liver metastases; MRI suggested SPN or PanNET	Referred to oncology center
Dec 2022	Laparoscopic partial hepatectomy	Liver metastasis: nested architecture, nuclear β-catenin^+^, Syn^+^, Ki-67 30%;	Pathology: “metastatic SPN”
Jan–Aug 2023	Systemic + targeted therapy (6 cycles)	Gemcitabine + S-1 + lenvatinib → pancreatic lesion shrunk to 2.3 cm; residual liver lesions 2.2 cm, stable	Partial response (PR)
Mar 2025	Laparoscopic distal pancreatectomy + RFA of liver metastases	3-cm hard pancreatic-tail mass adherent to peripancreatic fat;	R0 resection; uneventful recovery
Mar 2025	Final pathology & molecular testing	90% SPN (Ki-67 15%) + 10% PanNET G1 (Ki-67 1%);	Proven collision tumor
Apr 2025	Germline & tumor NGS	Somatic (Ex9-Int15); no CTNNB1/DAXX/ATRX/MEN1 mutations; germline negative	Molecularly proven APC mutation
Jan 2026	9-month post-operative imaging	no new lesions; lymph nodes 0.6 cm, unchanged	Continuous NED, asymptomatic

### Imaging findings

**Figure 1:**
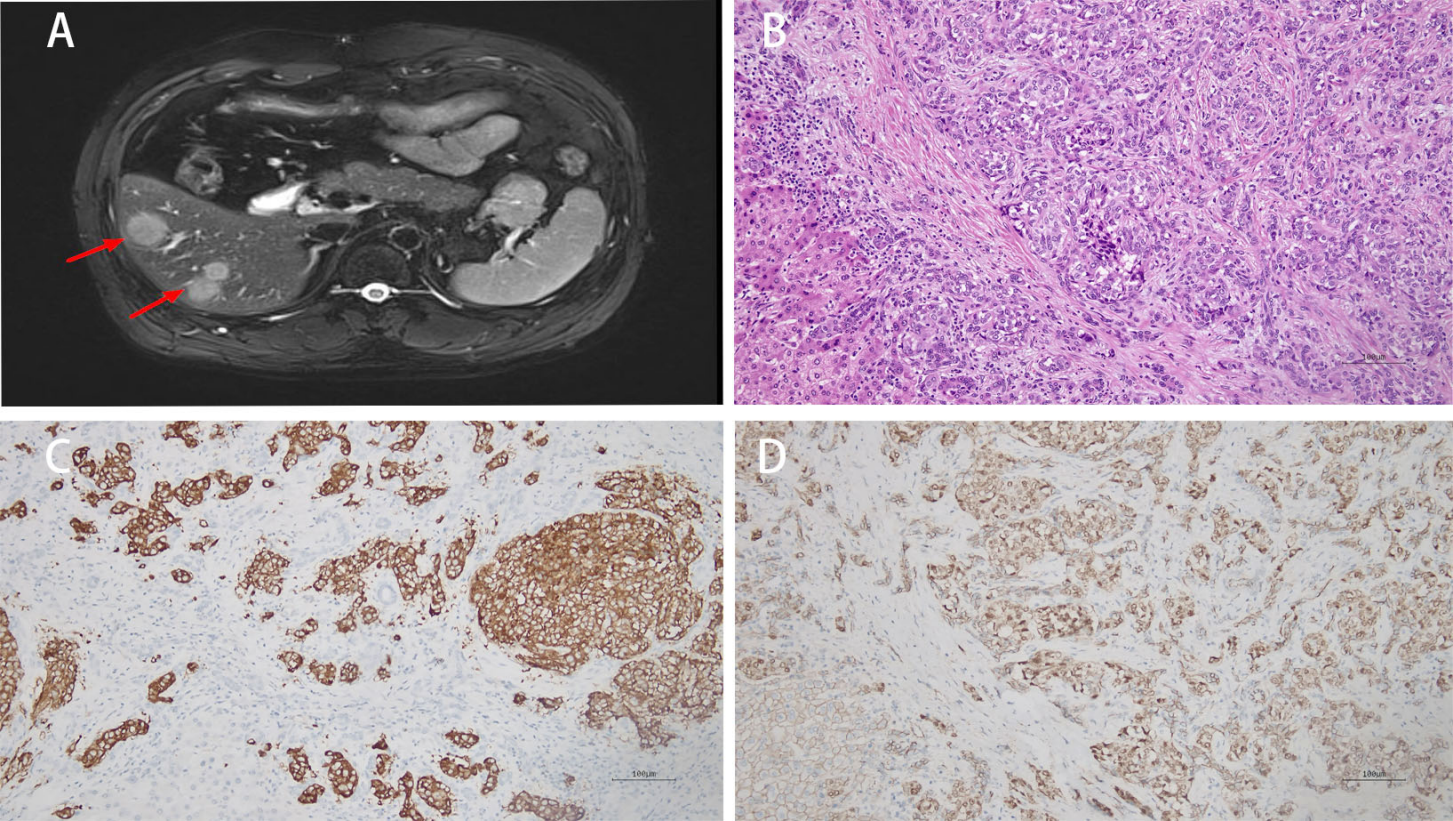
MRI shows multiple intrahepatic tumors (red arrows) **(A)**. The liver lesion displays infiltrative growth with a nested arrangement and cells showing vesicular nuclei (H&E, 200×) **(B)**. Tumor cells are positive for SYN (IHC, 200×) **(C)** and show nuclear β-catenin staining (IHC, 200×) **(D)**.

Contrast-enhanced 1.5-T MRI revealed a 4.2 × 3.4 × 3.3 cm lobulated exophytic mass with delayed enhancement in the pancreatic tail, as well as innumerable hepatic metastases, the largest of which measured 2.8 cm in segment VIII of the liver (**Figure 1A**).

### Initial management

The patient accepted a left-liver wedge resection, and the pathology of the liver lesion revealed a metastatic solid-pseudopapillary neoplasm (**Figure 1B**). The immunohistochemistry showed: SYN (+) (**Figure 1C**), nuclear β-catenin (+) (**Figure 1D**), Ki-67 30%; cGA, CD56, INSM1, and PR all negative. No PanNET component was identified in the hepatic specimen.

### Definitive surgery

After therapy, when the pancreatic mass was reduced to 2.3 cm, and the liver metastases were stable, the patient received a laparoscopic distal pancreatectomy with splenic preservation and radiofrequency ablation of dominant liver lesions.

### Pathology of the pancreatic mass

**Figure 2:**
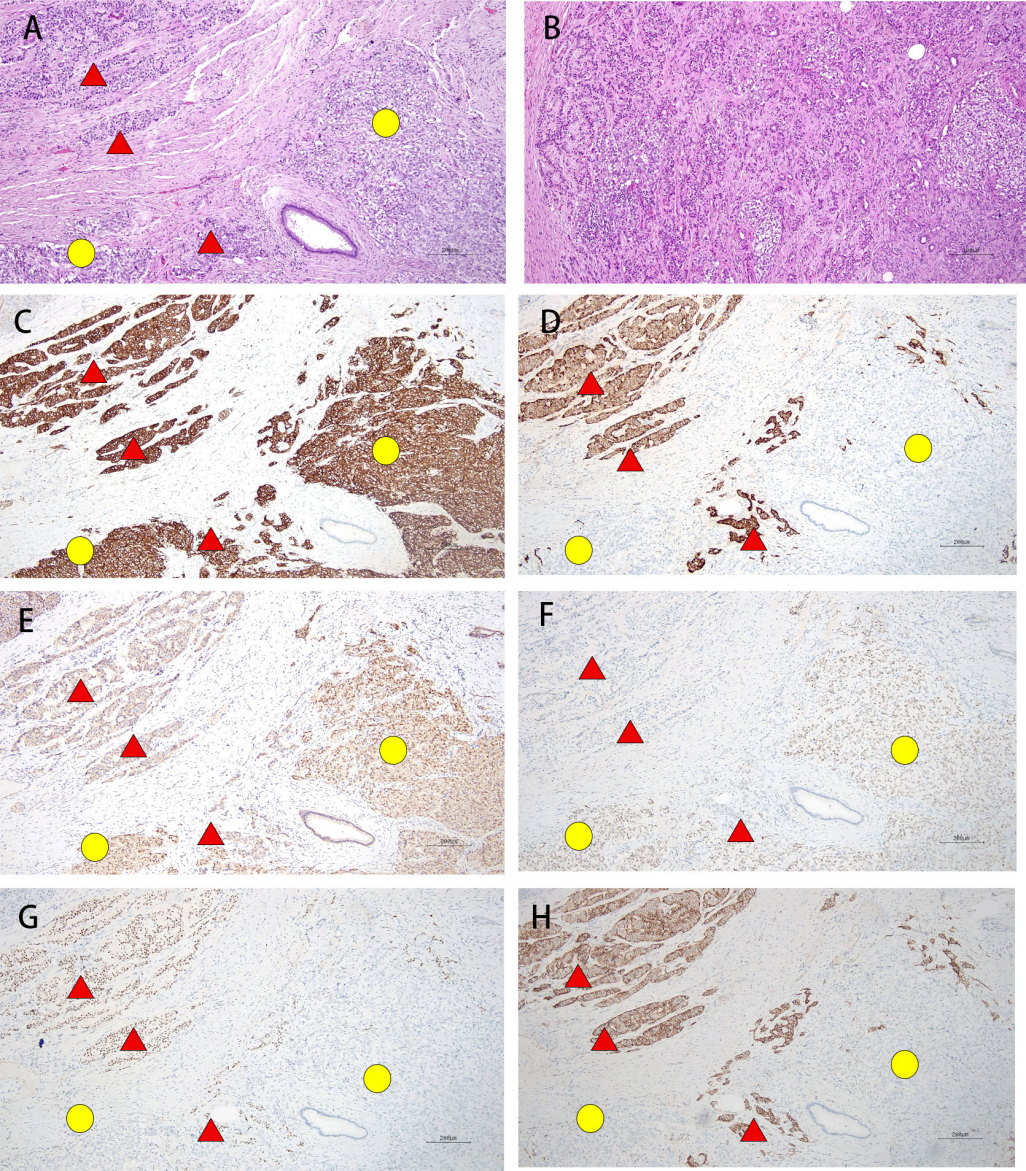
The pancreatic tumor contains two distinct components: a low-grade PanNET (upper-left and central-lower areas: indicated by red triangles) and a SPN (lower-right area: indicated by yellow circles) (H&E, 100×) **(A)**. The PanNET component shows a trabecular pattern with round, uniform cells, whereas the SPN component exhibits nested architecture, vesicular nuclei, and slightly clear cytoplasm; the two elements are intimately admixed yet distinct (H&E, 200×) **(B)**. Immunohistochemistry reveals diffuse, strong SYN positivity in both components (IHC, 100×) **(C)**. CgA is positive only in the PanNET component (indicated by red triangles) (IHC, 100×) **(D)**. β-catenin displays nuclear staining in the SPN areas (indicated by yellow circles) and membranous staining in the PanNET(indicated by red triangles) (IHC, 100×) **(E)**. LEF1 is nuclear in the SPN (indicated by yellow circles) and negative in the PanNET(indicated by red triangles) (IHC, 100×) **(F)**. INSM1 (IHC, 100×) **(G)** and SSTR2 (IHC, 100×) **(H)** are both positive exclusively in the PanNET (indicated by red triangles).

The resected pancreatic specimen revealed a 2.5 cm friable, ill-defined tumor with two distinct components (**Figures 2A, B**):

1. SPN (90%): Nested pattern, papillary structure is not obvious, poor cell adhesion, nuclear mild to moderate atypia, nuclear vacuoles, mitotic figure 2-3/2mm^2^. No definite necrosis was seen. AE1/AE3 (+), SYN (+) (**Figure 2C**), β-catenin (nuclear +) (**Figure 2E**), LEF-1 (+) (**Figure 2F**) and Ki-67 (15%); CD10 (-), PR (-), CgA (-) (**Figure 2D**), INSM1 (-) (**Figure 2G**) and SSTR2 (-) (**Figure 2H**).

2. PanNET G1 (10%): Island/trabecular pattern, the cells were round, small, and uniform, with mild atypia and mitotic figures of 0–1/2mm^2^. No definite necrosis was seen. AE1/AE3 (+), SYN (+) (**Figure 2C**), CgA (+) (**Figure 2D**), INSM1 (+) (**Figure 2G**), SSTR2 (3+) (**Figure 2H**), Ki-67 (1%), β-catenin (membranous +) (**Figure 2E**); CD10 (-), PR (-) and LEF-1 (-) (**Figure 2F**).

The two tumor components were mixed. Neural invasion was present; vascular invasion was absent; margins were negative.

Targeted next-generation sequencing (NGS) was performed on both the liver metastasis and the pancreatic primary tumor using a 520-gene cancer panel (~1.5 Mb genomic regions, ~1,000× mean coverage). Sequencing platform: Illumina NovaSeq 6000; Library preparation: Hybridization capture-based target enrichment. DNA source: Formalin-fixed paraffin-embedded (FFPE) tissue and peripheral blood (for germline testing).

The panel covers full coding sequences of key genes including APC (NM_000038.5, exons 1-15), CTNNB1 (NM_001904.3), MEN1 (NM_000244.3), DAXX (NM_001141970.1), and ATRX (NM_000489.4), as well as 23 genes tested for fusions (including ALK, BRAF, FGFR1/2/3, NTRK1/2/3, RET, ROS1, etc.). The complete gene list and RefSeq transcript accession numbers are provided in the **Supplementary Material**. Analysis revealed an in-frame APC rearrangement (Exon 9-Intron 15; APC-CNTNAP2 fusion); no mutations were detected in CTNNB1, MEN1, DAXX, ATRX, or other neuroendocrine-related genes. Germline testing using an 50-gene hereditary cancer panel (including APC gene, also listed in **Supplementary Material**) was negative.

## Follow-up

At 9-month follow-up, the patient remains asymptomatic with no evidence of disease progression. Imaging showed no new lesions, and lymph nodes remained stable at 0.6 cm. Given the APC alteration, elective gastroscopy and total colonoscopy were performed, revealing no definite gastric or intestinal lesions; regular gastrointestinal surveillance is ongoing.

## Discussion

### Origin of SPN vs. PanNET

Synchronous solid-pseudopapillary neoplasm (SPN) and pancreatic neuro-endocrine tumor (PanNET) are exceptionally rare; only 4 convincing cases have been described since 2015 (5–8). In some report the two primaries were separated by uninvolved parenchyma, lacked a histological transition zone, arguing against a “true” collision tumor, and almost all were discovered incidentally because the dominant SPN triggered imaging or surgery. Consequently, the PanNET—usually ≤25% of the aggregate volume and often labeled a “micro-adenoma”—is clinically silent.

Kosmahl’s embryonic-genital-ridge model (9) explains the classic SPN profile (nuclear β-catenin, PR(+), CgA (–)), whereas PanNETs arise from committed endocrine progenitors that retain membranous β-catenin and may harbor MEN1, DAXX or ATRX mutations (10, 11). These distinct molecular signatures support independent clonal origins rather than divergent differentiation from a shared precursor.

Our patient’s tumor pair was intimately admixed yet distinct in morphology and immunoprofile, however genomic analysis of both components identified only one driver event—an APC rearrangement previously reported in pure SPN. No CTNNB1, MEN1, DAXX/ATRX or germline variants were detected. The absence of additional mutations in the minor PanNET (10%) neither confirms nor refutes a collision origin. Larger series with multi-region sequencing are needed to clarify whether such composite neoplasms represent coincidence, shared predisposition, or an unrecognized hybrid pathway.

### Clinicopathologic overlap and diagnostic pitfalls between SPN and PanNET

Although SPN and PanNET differ in clinical presentation, histology, immunoprofile, and molecular drivers, their morphologic spectra frequently overlap. G2–G3 PanNETs may lose their organoid architecture and form solid sheets with nuclear pleomorphism and brisk mitosis, mimicking the monotonous, loosely cohesive cells of SPN (12). Conversely, SPN can shed its classic pseudopapillae and hyaline globules, adopting a compact nested or trabecular pattern indistinguishable from a neuro-endocrine neoplasm (13). Immunohistochemistry is therefore mandatory, yet the traditional “CgA-positive equals PanNET” rule is unreliable: 31% of well-differentiated PanNETs are CgA-negative (14), and rare cases show aberrant nuclear β-catenin (15), further muddying the waters.

Our collision tumor illustrates a practical, literature-supported antibody panel that restores clarity: (1) INSM1 – diffusely positive in the PanNET (G1) component, completely negative in adjacent SPN. (2) SSTR2 – strong membranous staining in PanNET, absent in SPN. (3) LEF-1 – nuclear expression restricted to SPN, absent in PanNET, confirming Wnt-pathway activation with high specificity (16). (4) β-catenin localization – diffuse nuclear in SPN, strictly membranous in PanNET.

Recent large-scale studies have identified ABCD1 as a highly sensitive and specific positive marker for SPN (17); diffuse cytoplasmic ABCD1 staining (+++/++) is present in > 95% of SPNs, while being negative or only weakly positive in PanNETs. Adding ABCD1 to the above panel, therefore: (1) provides a positive “SPN signal” when β-catenin is equivocal or patchy (e.g., in small biopsies); and (2) reduces false-negative diagnoses in metastatic sites where PanNET-like morphology may predominate. Thus, for any pancreatic tumor with ambiguous morphology, limited material, or equivocal β-catenin staining, a four-marker combination of ABCD1, INSM1, SSTR2, and LEF-1 (with β-catenin localization) offers a rapid, reproducible algorithm to separate SPN from PanNET and directs the decision for confirmatory genomic testing.

### APC-mutated SPN – clinicopathologic peculiarities

APC-mutated solid-pseudopapillary neoplasms (SPNs) represent a rare molecular subset, accounting for <10% of cases (18, 19). In this case, a genomic rearrangement involving APC was identified that spans Exon 9 to Intron 15. This rearrangement simultaneously fuses the 5′ end of APC (containing the promoter and the first several exons) with a segment of the neighboring CNTNAP2 gene, generating a chimeric transcript. Exon 15 of APC comprises more than 60% of the coding sequence and is a mutational hotspot; the rearrangement causes a frameshift or premature termination, yielding a truncated protein that is unable to assemble the “destruction complex” with Axin/GSK-3β. So β-catenin cannot be phosphorylated and degraded. Consequently, β-catenin accumulates in the cytoplasm and nucleus.

A limitation of this study is that the APC rearrangement was identified by targeted NGS alone and was not validated by an independent method such as Sanger sequencing or fluorescence *in situ* hybridization (FISH). However, the NGS data showed high read depth and clear breakpoint identification, and the identical fusion was detected in both the primary pancreatic tumor and the liver metastasis, providing internal consistency. Future studies should include orthogonal validation of rearrangements identified by NGS.

Across the seven previously documented cases of APC-driven solid-pseudopapillary neoplasms and our present case (**Table 2**), the classic “young-woman” profile disappears. Sex distribution is equal (4 M: 4 F), and median age diverges sharply: 46 years in men versus 25 years in women. Five tumors (63%) arose in proven or inferred FAP kindreds; three were confirmed germline, two were deduced from pedigree and extracolonic lesions, underscoring that an SPN may be the first clinical clue to hereditary APC loss. Morphology and immunophenotype remain indistinguishable from CTNNB1-mutant examples: every lesion displayed solid-cystic architecture, uniform grooved nuclei, and diffuse nuclear β-catenin with CD10/PR/cyclin-D1 co-expression. Nevertheless, the outcome is less benign: two men developed liver metastases, and one died, while the index patient (M/34) showed both neural invasion and a synchronous 10% G1-PanNET collision component—the only such composite reported to date. It should be noted that the current follow-up duration for this patient remains relatively short (9 months), and no adverse signs have been observed to date. However, given the “less benign” nature of APC-mutated SPNs and the coexistence of a synchronous PanNET, longer-term surveillance is warranted to fully assess metastatic potential and detect any late recurrence. Thus, APC-mutated SPNs are sexually balanced, frequently FAP-associated, histologically familiar, yet retain genuine metastatic potential; identification mandates germline testing, intestinal surveillance, and complete oncologic resection even for small masses.

**Table 2:** Literature review of all eight documented APC-driven SPN (seven previously reported cases and the present case).

Ref.	Sex/age(year)	Site of pancreas	Size	Key morphology	APC molecular defect	FAP	Key immunoprofile	Follow-up/prognosis
Jayson Wang, 2018 (4)	M/64	Not available	Not available	Circumscribed mass with necrosis and focal nuclear pleomorphism	c.3964delG p.(Glu1322Lysfs93)	Not mention	β-catenin nuclear+	24mo, liver metastasis, and died
Naoi 2021 (20)	F/20	Tail	10 cm	Solid-cystic, calcified capsule, no malignant features	Promoter 1A + 1B copy-number loss (MLPA)	Y	β-catenin nuclear+, Chromogranin–, Synaptophysin–	No recurrence 5 y after distal pancreatectomy
Meira-Júnior 2022 (case1) (21)	M/54	Tail	1.2 cm	Unencapsulated, hemorrhagic foci	Inferred from clinical presentation without sequencing results	Y	β-catenin nuclear+, Cyclin-D1 nuclear+	No recurrence (follow-up not specified)
Meira-Júnior 2022 (case2) (21)	F/34	Neck	2.4 cm	heterogenic mass	Inferred from clinical presentation without sequencing results	Y	β-catenin nuclear+	No recurrence (follow-up not specified)
El Halabi 2023 (22)	F/25	Head	7.0 cm	Solid with central necrosis, abutment of SMV & artery	Inferred from the pedigree, germline IVS4 + 2insT (Her mother)	Y	β-catenin nuclear+, CD10+, Chromogranin–	Disease-free 40 mo after pancreaticoduodenectomy
A. Sciarra 2024 (23)	M/38	Body-tail junction	4.0 cm	Not available	Germline pathogenic variant c.2677G>T p.(Glu893Ter)	Y	β-catenin nuclear+, CD10+, Cyclin-D1 nuclear+, Sox-11+, PR+, E-cadherin abnormal (cytoplasmic), Neuroendocrine markers-	Not available
Arshad 2025 (24)	F/33	Head + Tail (multifocal)	2.3 cm & 1.4 cm	Two discrete SPNs, solid-cystic	Exons 6–16 deletion	Y	β-catenin nuclear+, PR+, CD10+	On surveillance, no progression reported
Present case(this study)	M/34	Tail (collision tumor: 90% SPN + 10% PanNET G1)	2.5 cm	SPN component: Ki-67 15%, neural invasion, liver metastases	Somatic APC rearrangement (Exon9_Intron15; APC-CNTNAP2)	N	SPN: β-catenin nuclear+, LEF-1+; PanNET: membranous β-catenin, CgA+, INSM1+	9 mo post distal pancreatectomy + RFA: no new lesions

mo, month(s); FAP, Familial Adenomatous Polyposis; SMV, superior mesenteric vein; RFA, radiofrequency ablation.

## Conclusion

We present the first molecularly confirmed case of a synchronous solid-pseudopapillary neoplasm (SPN) and low-grade pancreatic neuroendocrine tumor (PanNET, G1) forming a collision tumor, driven by an APC gene rearrangement rather than the canonical CTNNB1 mutation. This case underscores the diagnostic complexity posed by morphologically overlapping pancreatic neoplasms and highlights the critical role of comprehensive immunohistochemical profiling and genomic characterization in achieving accurate classification. The identification of an APC alteration not only redefines the molecular underpinnings of this rare collision tumor but also suggests a potential predisposing role of APC loss in facilitating multi-clonal tumorigenesis. Given the aggressive phenotype observed in APC-mutated SPNs, including metastatic potential and possible association with hereditary cancer syndromes such as FAP, we advocate for germline testing and long-term surveillance in affected patients. This report expands the molecular spectrum of SPN and reinforces the importance of integrating histologic, immunophenotypic, and genetic data in the diagnostic workup of complex pancreatic tumors.

## Data Availability

The original contributions presented in the study are included in the article/**Supplementary Material**. Further inquiries can be directed to the corresponding author.
