# Supplementary material for: APC-rearranged solid pseudopapillary neoplasm–pancreatic neuroendocrine tumor collision tumor: a molecularly resolved case report
Source: Front Oncol. 2026 May 7;16:1796302. doi: 10.3389/fonc.2026.1796302 (PMC13195583; doi:10.3389/fonc.2026.1796302)
Supplement: Supplementary file 1 [file Table1.docx]

**Attachment**

The list of genes analyzed by NGS:

| ABL1 NM_005157.5 | ABL2 NM_007314.3 | ABRAXAS1 NM_139076.2 | ACVR1 NM_001105.4 | ACVR1B NM_020328.3 |
| --- | --- | --- | --- | --- |
| AKT1 NM_001014432.1 | AKT2 NM_001626.5 | AKT3 NM_005465.4 | ALK NM_004304.4 | ALOX12B NM_001139.2 |
| AMER1 NM_152424.3 | APC NM_000038.5 | AR NM_000044.3 | ARAF NM_001256196.1 | ARFRP1 NM_001267547.2 |
| ARID1A NM_006015.4 | ARID1B NM_020732.3 | ARID2 NM_152641.2 | ARID5B NM_032199.2 | ASXL1 NM_015338.5 |
| ASXL2 NM_018263.4 | ATM NM_000051.3 | ATR NM_001184.3 | ATRX NM_000489.4 | AURKA NM_001323303.1 |
| AURKB NM_001284526.1 | AXIN1 NM_003502.3 | AXIN2 NM_004655.3 | AXL NM_021913.4 | B2M NM_004048.2 |
| BAP1 NM_004656.3 | BARD1 NM_000465.3 | BBC3 NM_001127240.2 | BCL10 NM_003921.4 | BCL2 NM_000633.2 |
| BCL2L1 NM_001317919.1 | BCL2L11 NM_001204107.1 | BCL2L2 NM_001199839.1 | BCL6 NM_001130845.1 | BCOR NM_001123383.1 |
| BCORL1 NM_021946.4 | BIRC3 NM_001165.4 | BLM NM_000057.3 | BMPR1A NM_004329.2 | BRAF NM_004333.4 |
| BRCA1 NM_007294.3 | BRCA2 NM_000059.3 | BRD4 NM_058243.2 | BRD7 NM_001173984.2 | BRINP3 NM_199051.2 |
| BRIP1 NM_032043.2 | BTG1 NM_001731.2 | BTG2 NM_006763.2 | BTK NM_000061.2 | CALR NM_004343.3 |
| CARD11 NM_032415.5 | CASP8 NM_001228.4 | CBFB NM_022845.2 | CBL NM_005188.3 | CCND1 NM_053056.2 |
| CCND2 NM_001759.3 | CCND3 NM_001760.4 | CCNE1 NM_001238.3 | CD274 NM_014143.3 | CD74 NM_001025159.2 |
| CD79A NM_001783.3 | CD79B NM_000626.3 | CDC73 NM_024529.4 | CDH1 NM_004360.4 | CDH18 NM_001291956.1 |
| CDK12 NM_016507.3 | CDK4 NM_000075.3 | CDK6 NM_001145306.1 | CDK8 NM_001260.2 | CDKN1A NM_001291549.1 |
| CDKN1B NM_004064.4 | CDKN1C NM_000076.2 | CDKN2A NM_000077.4 | CDKN2B NM_004936.3 | CDKN2C NM_001262.2 |
| CEBPA NM_004364.4 | CENPA NM_001809.3 | CHD1 NM_001270.2 | CHD2 NM_001271.3 | CHD4 NM_001273.3 |
| CHEK1 NM_001274.5 | CHEK2 NM_007194.3 | CIC NM_015125.4 | CREBBP NM_004380.2 | CRKL NM_005207.3 |
| CRLF2 NM_022148.3 | CSF1R NM_001288705.1 | CSF3R NM_156039.3 | CSMD1 NM_033225.5 | CSMD3 NM_198123.1 |
| CTCF NM_006565.3 | CTLA4 NM_005214.4 | CTNNA1 NM_001323982.1 | CTNNB1 NM_001904.3 | CUL3 NM_001257198.1 |
| CUL4A NM_001008895.2 | CXCR4 NM_003467.2 | CYLD NM_015247.2 | CYP17A1 NM_000102.3 | CYP2D6 NM_000106.5 |
| DAXX NM_001141970.1 | DCUN1D1 NM_020640.3 | DDR1 NM_013994.2 | DDR2 NM_001014796.1 | DICER1 NM_177438.2 |
| DIS3 NM_014953.4 | DNAJB1 NM_006145.2 | DNMT1 NM_001130823.2 | DNMT3A NM_022552.4 | DNMT3B NM_006892.3 |
| DOT1L NM_032482.2 | DPYD NM_000110.3 | EED NM_001308007.1 | EGFR NM_005228.3 | EIF1AX NM_001412.3 |
| EIF4E NM_001130679.1 | EMSY NM_001300942.1 | EP300 NM_001429.3 | EPCAM NM_002354.2 | EPHA2 NM_004431.4 |
| EPHA3 NM_005233.5 | EPHA5 NM_001281765.2 | EPHA7 NM_004440.3 | EPHB1 NM_004441.4 | EPHB4 NM_004444.4 |
| ERBB2 NM_004448.3 | ERBB3 NM_001982.3 | ERBB4 NM_005235.2 | ERCC1 NM_202001.2 | ERCC2 NM_000400.3 |
| ERCC3 NM_000122.1 | ERCC4 NM_005236.2 | ERCC5 NM_000123.3 | ERG NM_001136154.1 | ERRFI1 NM_018948.3 |
| ESR1 NM_000125.3 | ETV4 NM_001079675.2 | ETV5 NM_004454.2 | ETV6 NM_001987.4 | EWSR1 NM_013986.3 |
| EZH2 NM_004456.4 | EZR NM_001111077.1 | FANCA NM_000135.2 | FANCC NM_000136.2 | FANCD2 NM_001018115.2 |
| FANCE NM_021922.2 | FANCF NM_022725.3 | FANCG NM_004629.1 | FANCI NM_001113378.1 | FANCL NM_018062.3 |
| FANCM NM_020937.3 | FAS NM_000043.5 | FAT1 NM_005245.3 | FBXW7 NM_033632.3 | FGF10 NM_004465.1 |
| FGF12 NM_021032.4 | FGF14 NM_175929.2 | FGF19 NM_005117.2 | FGF23 NM_020638.2 | FGF3 NM_005247.2 |
| FGF4 NM_002007.2 | FGF6 NM_020996.2 | FGF7 NM_002009.3 | FGFR1 NM_023110.2 | FGFR2 NM_000141.4 |
| FGFR3 NM_000142.4 | FGFR4 NM_002011.4 | FH NM_000143.3 | FLCN NM_144997.5 | FLT1 NM_002019.4 |
| FLT3 NM_004119.2 | FLT4 NM_182925.4 | FOXA1 NM_004496.3 | FOXL2 NM_023067.3 | FOXO1 NM_002015.3 |
| FOXP1 NM_001244810.1 | FRS2 NM_001042555.2 | FUBP1 NM_003902.4 | FYN NM_002037.5 | GABRA6 NM_000811.2 |
| GATA1 NM_002049.3 | GATA2 NM_001145661.1 | GATA3 NM_001002295.1 | GATA4 NM_001308093.1 | GATA6 NM_005257.5 |
| GEN1 NM_001130009.2 | GID4 NM_024052.4 | GLI1 NM_005269.2 | GNA11 NM_002067.4 | GNA13 NM_006572.5 |
| GNAQ NM_002072.4 | GNAS NM_080425.3 | GPS2 NM_004489.4 | GREM1 NM_013372.6 | GRIN2A NM_000833.4 |
| GRM3 NM_000840.2 | GSK3B NM_002093.3 | H3C2 NM_003537.3 | H3F3A NM_002107.4 | H3F3B NM_005324.4 |
| H3F3C NM_001013699.2 | HDAC1 NM_004964.2 | HDAC2 NM_001527.3 | HGF NM_000601.5 | HIST1H1C NM_005319.3 |
| HIST1H2BD NM_021063.3 | HIST1H3A NM_003529.2 | HIST1H3C NM_003531.2 | HIST1H3D NM_003530.4 | HIST1H3E NM_003532.2 |
| HIST1H3G NM_003534.2 | HIST1H3H NM_003536.2 | HIST1H3I NM_003533.2 | HIST1H3J NM_003535.2 | HIST2H3D NM_001123375.2 |
| HIST3H3 NM_003493.2 | HLA-A NM_001242758.1 | HLA-B NM_005514.7 | HLA-C NM_001243042.1 | HNF1A NM_000545.6 |
| HOXB13 NM_006361.5 | HRAS NM_005343.3 | HSD3B1 NM_000862.2 | HSP90AA1 NM_001017963.2 | ICOSLG NM_001283050.1 |
| ID3 NM_002167.4 | IDH1 NM_005896.3 | IDH2 NM_002168.3 | IFNGR1 NM_000416.2 | IGF1 NM_001111285.2 |
| IGF1R NM_000875.4 | IGF2 NM_000612.5 | IKBKE NM_014002.3 | IKZF1 NM_006060.5 | IL10 NM_000572.2 |
| IL7R NM_002185.3 | INHA NM_002191.3 | INHBA NM_002192.3 | INPP4A NM_001134224.1 | INPP4B NM_001101669.1 |
| INSR NM_000208.3 | IRF2 NM_002199.3 | IRF4 NM_002460.3 | IRS1 NM_005544.2 | IRS2 NM_003749.2 |
| JAK1 NM_001320923.1 | JAK2 NM_004972.3 | JAK3 NM_000215.3 | JUN NM_002228.3 | KAT6A NM_006766.4 |
| KDM5A NM_001042603.2 | KDM5C NM_004187.3 | KDM6A NM_001291415.1 | KDR NM_002253.2 | KEAP1 NM_012289.3 |
| KEL NM_000420.2 | KIT NM_000222.2 | KLF4 NM_001314052.1 | KLHL6 NM_130446.2 | KMT2A NM_001197104.1 |
| KMT2C NM_170606.2 | KMT2D NM_003482.3 | KRAS NM_033360.3 | LATS1 NM_004690.3 | LATS2 NM_014572.2 |
| LMO1 NM_002315.2 | LRP1B NM_018557.2 | LTK NM_002344.5 | LYN NM_002350.3 | MAF NM_005360.4 |
| MAGI2 NM_012301.3 | MALT1 NM_006785.3 | MAP2K1 NM_002755.3 | MAP2K2 NM_030662.3 | MAP2K4 NM_001281435.1 |
| MAP3K1 NM_005921.1 | MAP3K13 NM_001242314.1 | MAPK1 NM_002745.4 | MAPK3 NM_002746.2 | MAX NM_002382.4 |
| MCL1 NM_021960.4 | MDC1 NM_014641.2 | MDM2 NM_002392.5 | MDM4 NM_002393.4 | MED12 NM_005120.2 |
| MEF2B NM_001145785.1 | MEN1 NM_000244.3 | MERTK NM_006343.2 | MET NM_000245.3 | MGA NM_001164273.1 |
| MIR21 NR_029493.1 | MITF NM_000248.3 | MKNK1 NM_003684.5 | MLH1 NM_000249.3 | MLH3 NM_001040108.1 |
| MPL NM_005373.2 | MRE11 NM_005591.3 | MSH2 NM_000251.2 | MSH3 NM_002439.4 | MSH6 NM_000179.2 |
| MST1 NM_020998.3 | MST1R NM_002447.3 | MTAP NM_002451.3 | MTOR NM_004958.3 | MUTYH NM_001128425.1 |
| MYC NM_002467.4 | MYCL NM_001033082.2 | MYCN NM_001293228.1 | MYD88 NM_002468.4 | MYOD1 NM_002478.4 |
| NAV3 NM_001024383.1 | NBN NM_002485.4 | NCOA3 NM_181659.2 | NCOR1 NM_006311.3 | NCOR2 NM_006312.5 |
| NEGR1 NM_173808.2 | NF1 NM_000267.3 | NF2 NM_000268.3 | NFE2L2 NM_006164.4 | NFKBIA NM_020529.2 |
| NKX2-1 NM_001079668.2 | NKX3-1 NM_006167.3 | NOTCH1 NM_017617.4 | NOTCH2 NM_024408.3 | NOTCH3 NM_000435.2 |
| NOTCH4 NM_004557.3 | NPM1 NM_002520.6 | NRAS NM_002524.4 | NRG1 NM_001322205.1 | NSD1 NM_022455.4 |
| NSD2 NM_001042424.2 | NSD3 NM_023034.1 | NT5C2 NM_001134373.2 | NTHL1 NM_002528.6 | NTRK1 NM_001007792.1 |
| NTRK2 NM_006180.4 | NTRK3 NM_001012338.2 | NUP93 NM_014669.4 | NUTM1 NM_001284292.1 | P2RY8 NM_178129.4 |
| PAK1 NM_001128620.1 | PAK3 NM_001128168.2 | PAK5 NM_020341.3 | PALB2 NM_024675.3 | PARP1 NM_001618.3 |
| PARP2 NM_005484.3 | PARP3 NM_001003931.3 | PAX5 NM_016734.2 | PBRM1 NM_018313.4 | PCDH11X NM_032968.4 |
| PDCD1 NM_005018.2 | PDCD1LG2 NM_025239.3 | PDGFRA NM_006206.4 | PDGFRB NM_002609.3 | PDK1 NM_001278549.1 |
| PGR NM_000926.4 | PHOX2B NM_003924.3 | PIK3C2B NM_002646.3 | PIK3C2G NM_001288772.1 | PIK3C3 NM_002647.3 |
| PIK3CA NM_006218.3 | PIK3CB NM_006219.2 | PIK3CD NM_005026.3 | PIK3CG NM_001282426.1 | PIK3R1 NM_181523.2 |
| PIK3R2 NM_005027.3 | PIK3R3 NM_001303427.1 | PIM1 NM_001243186.1 | PLCG2 NM_002661.4 | PLK2 NM_006622.3 |
| PMS1 NM_000534.4 | PMS2 NM_000535.6 | PNRC1 NM_006813.2 | POLD1 NM_001256849.1 | POLE NM_006231.3 |
| PPARG NM_015869.4 | PPM1D NM_003620.3 | PPP2R1A NM_014225.5 | PPP2R2A NM_002717.3 | PPP6C NM_001123355.1 |
| PRDM1 NM_001198.3 | PREX2 NM_024870.3 | PRKAR1A NM_002734.4 | PRKCI NM_002740.5 | PRKDC NM_006904.6 |
| PRKN NM_004562.2 | PTCH1 NM_000264.3 | PTEN NM_000314.6 | PTPN11 NM_002834.3 | PTPRD NM_002839.3 |
| PTPRO NM_030667.2 | PTPRS NM_002850.3 | PTPRT NM_133170.3 | QKI NM_006775.2 | RAB35 NM_006861.6 |
| RAC1 NM_018890.3 | RAD21 NM_006265.2 | RAD50 NM_005732.3 | RAD51 NM_001164269.1 | RAD51B NM_133509.3 |
| RAD51C NM_058216.2 | RAD51D NM_002878.3 | RAD52 NM_001297419.1 | RAD54L NM_003579.3 | RAF1 NM_002880.3 |
| RARA NM_000964.3 | RASA1 NM_002890.2 | RB1 NM_000321.2 | RBM10 NM_001204468.1 | RECQL4 NM_004260.3 |
| REL NM_002908.3 | RET NM_020975.4 | RHEB NM_005614.3 | RHOA NM_001664.3 | RICTOR NM_001285439.1 |
| RIT1 NM_001256821.1 | RNF43 NM_017763.5 | ROS1 NM_002944.2 | RPA1 NM_002945.3 | RPS6KA4 NM_003942.2 |
| RPS6KB2 NM_003952.2 | RPTOR NM_020761.2 | RSPO2 NM_178565.4 | RUNX1 NM_001754.4 | RUNX1T1 NM_001198679.1 |
| SDC4 NM_002999.3 | SDHA NM_004168.3 | SDHAF2 NM_017841.2 | SDHB NM_003000.2 | SDHC NM_003001.3 |
| SDHD NM_003002.3 | SETD2 NM_014159.6 | SF3B1 NM_012433.3 | SGK1 NM_001143676.1 | SH2B3 NM_005475.2 |
| SH2D1A NM_002351.4 | SHQ1 NM_018130.2 | SLC34A2 NM_006424.2 | SLIT2 NM_004787.3 | SLX4 NM_032444.2 |
| SMAD2 NM_001003652.3 | SMAD3 NM_005902.3 | SMAD4 NM_005359.5 | SMARCA4 NM_001128849.1 | SMARCB1 NM_003073.4 |
| SMARCD1 NM_003076.4 | SMO NM_005631.4 | SNCAIP NM_001308100.1 | SOCS1 NM_003745.1 | SOX10 NM_006941.3 |
| SOX17 NM_022454.3 | SOX2 NM_003106.3 | SOX9 NM_000346.3 | SPEN NM_015001.2 | SPOP NM_001007226.1 |
| SPTA1 NM_003126.2 | SRC NM_198291.2 | SRSF2 NM_003016.4 | STAG2 NM_001042749.2 | STAT3 NM_139276.2 |
| STAT4 NM_001243835.1 | STAT5A NM_001288718.1 | STAT5B NM_012448.3 | STK11 NM_000455.4 | STK40 NM_001282546.1 |
| SUFU NM_016169.3 | SYK NM_001174167.2 | TAF1 NM_001286074.1 | TBX3 NM_016569.3 | TCF3 NM_003200.3 |
| TCF7L2 NM_001146274.1 | TEK NM_000459.4 | TENT5C NM_017709.3 | TERC NR_001566.1 | TERT NM_198253.2 |
| TET1 NM_030625.2 | TET2 NM_001127208.2 | TGFBR1 NM_001306210.1 | TGFBR2 NM_001024847.2 | TIPARP NM_001184717.1 |
| TMEM127 NM_017849.3 | TMPRSS2 NM_001135099.1 | TNFAIP3 NM_001270507.1 | TNFRSF14 NM_003820.3 | TOP1 NM_003286.2 |
| TOP2A NM_001067.3 | TP53 NM_000546.5 | TP63 NM_003722.4 | TRAF2 NM_021138.3 | TRAF7 NM_032271.2 |
| TRIM58 NM_015431.3 | TRPC5 NM_012471.2 | TSC1 NM_000368.4 | TSC2 NM_000548.4 | TSHR NM_000369.2 |
| TYRO3 NM_006293.3 | U2AF1 NM_001025203.1 | UGT1A1 NM_000463.2 | VEGFA NM_001025366.2 | VEGFB NM_003377.4 |
| VHL NM_000551.3 | WISP3 NM_198239.1 | WRN NM_000553.4 | WT1 NM_024426.4 | XIAP NM_001167.3 |
| XPO1 NM_003400.3 | XRCC2 NM_005431.1 | XRCC3 NM_001100118.1 | YAP1 NM_001282101.1 | YES1 NM_005433.3 |
| ZBTB16 NM_001018011.1 | ZBTB2 NM_020861.2 | ZNF217 NM_006526.2 | ZNF703 NM_025069.2 | ZNRF3 NM_001206998.1 |

Note: The NM accession number following each gene name indicates the RefSeq transcript used for annotation.

Genes simultaneously tested for fusion:

| ALK | BRAF | CD274 | CD74 | ETV4 | ETV5 |
| --- | --- | --- | --- | --- | --- |
| ETV6 | EWSR1 | EZR | FGFR1 | FGFR2 | FGFR3 |
| NRG1 | NTRK1 | NTRK2 | NTRK3 | RAF1 | RET |
| ROS1 | RSPO2 | SDC4 | SLC34A2 | TMPRSS2 |  |

Germline Genetic Testing Gene Panel：

| APC | ATM | AXIN2 | BAP1 | BARD1 | BLM | BMPR1A |
| --- | --- | --- | --- | --- | --- | --- |
| CDC73 | CDH1 | CDK12 | CDK4 | CDKN1B | CDKN2A | CHEK1 |
| EPCAM | FANCA | FANCI | FANCL | FH | FLCN | GREM1 |
| MEN1 | MET | MLH1 | MSH2 | MSH3 | MSH6 | MUTYH |
| NTHL1 | PALB2 | PDGFRA | PMS2 | POLD1 | POLE | PPP2R2A |
| RAD51B | RAD51C | RAD51D | RAD54L | RB1 | RET | SDHA |
| SDHD | SMAD4 | SMARCA4 | SMARCB1 | STK11 | TMEM127 | TP53 |
| WT1 |  |  |  |  |  |  |
